# Mast cell activation syndrome-related unusual periocular inflammation associated with mydriatic eyedrops during cataract surgery^☆^

**DOI:** 10.1016/j.ajoc.2026.102588

**Published:** 2026-04-24

**Authors:** Anny M.S. Cheng, Aishah Ali, John T. LiVecchi, Hailey Mair, Elizabeth S. Yang, Jade Gieseke Guevara

**Affiliations:** a Department of Ophthalmology, University of Florida, Gainesville, FL, USA; b Department of Ophthalmology, Florida International University, Herbert Wertheim College of Medicine, Miami, FL, USA; c Department of Internal Medicine, Allergist and Immunologist, Washington DC, USA; d New York University, College of Arts and Sciences, New York, NY, USA; e Insight Eye Institute, Lighthouse Point, FL, USA; f Department of Ophthalmology, Broward Health, Fort Lauderdale, FL, USA

**Keywords:** Allergy, Cataract, Mast cell activation syndrome, Periocular inflammatory, Steroid

## Abstract

**Purpose:**

Mast Cell Activation Syndrome (MCAS) is a condition characterized by the inappropriate and unpredictable activation of mast cells, resulting in episodic symptoms affecting various parts of the body. Cutaneous, gastrointestinal, respiratory, and cardiovascular symptoms are often reported and well characterized in MCAS. However, ocular manifestations associated with this condition are not frequently explored.

**Observations:**

This case report describes an unusual periocular inflammatory reaction in a 76-year-old male with a history of MCAS following uneventful cataract surgery, specifically triggered by procedural mydriatic eyedrops (tropicamide). The patient, diagnosed with MCAS based on symptoms, elevated tryptase, and adequate response to therapies, presented to ophthalmology clinic with pre-existing dry eye disease and blepharitis for blurred vision. Despite prophylactic treatment with oral and intravenous (IV) antihistamines, intraoperative subconjunctival dexamethasone, and postoperative anti-inflammatory medications, the patient developed periocular swelling and erythema, on the first postoperative day, following uneventful cataract surgery during which intraprocedural tropicamide was administered for dilation. Importantly, the reaction may have been triggered not by tropicamide itself but by one or more excipients (e.g., benzalkonium chloride) known to induce mast cell activation in susceptible individuals.

**Conclusions and importance:**

This unusual presentation, previously not well described in the context of MCAS, highlights the critical need for ophthalmologists to recognize the heightened risk of severe periocular inflammation in MCAS patients undergoing procedures involving routine mydriatic agents. The case underscores that such reactions may be excipient-driven rather than drug-specific, emphasizes the complex interplay between MCAS, ocular surface conditions, and ophthalmic medications, and necessitates increased awareness among healthcare providers to tailor management to prevent potentially severe outcomes.

## 1 Introduction

Mast Cell Activation syndrome (MCAS) is a disorder characterized by the severe, episodic, and recurrent symptoms induced by inappropriate mast cell activation.^1^ Although previously underrecognized, MCAS is increasingly recognized as a heterogeneous and often underdiagnosed condition. Ocular symptoms, including eye irritation, have been reported as common ad as part of the multisystem presentation in a large MCAS population^2^; however, there are limited reports of isolated ocular symptoms or drug induced ocular reactions in MCAS. There are reports of ocular involvement in mastocytosis, a mast cell disease distinct from MCAS, in which mast cells proliferate and infiltrate organs.^3^ A French hospital-based single-center study in mastocytosis patients found that 81% experienced one or more eye symptoms, with ocular itching, dryness, and excessive tearing being the most frequently reported.^3^ Other case reports have documented redness, irritation of the sclera, eyelid, and conjunctivitis that can affect one or both eyes. Herein, we described a case focused on an unusual post-operative periocular inflammatory reaction in a patient with MCAS following an uneventful ophthalmic cataract procedure. While allergic contact dermatitis or other hypersensitivity to ophthalmic medications is uncommon,^4^ this specific case highlights the heightened risk and complexity when MCAS is present. After conducting a literature review on July 20,2025 utilizing PubMed, Google Scholar, Ovid, and EBSCO using the key words (mast cell activation syndrome, eye, ocular), we did not find any prior reports of periocular inflammatory reaction.

## 2 Case

A 76-year-old man with a history of chronic diarrhea and maculopapular skin lesions on his limbs and trunk was referred for evaluation of a cataract in his right eye. He was diagnosed with MCAS after multidisciplinary (allergy, dermatology, hematology, and rheumatology) evaluation, based on characteristic multisystem symptoms, exclusion of alternative diagnoses, and laboratory findings suggestive of mast cell involvement, including elevated serum tryptase levels during a period of active skin symptoms (but with negative skin biopsies for mast cells). Additionally, he had experienced multiple episodes of periocular erythema and swelling following the application of dilating drops; however, the details are unclear per the patient and lack of accessibility to prior patient records.

He presented to ophthalmology clinic with a right eye blurred vision associated with bilateral foreign body sensation and dryness. Upon examination, the patient's vision was 20/50 in the right eye and 20/25 in the left eye with bilateral collarettes of the upper and lower eyelids. Both eyes showed mild interpalpebral conjunctival injection, a scant tear meniscus, and a reduced Schirmer test result of 5 mm. Slit-lamp examination revealed nuclear and posterior capsular cataract in the right eye more than in the left eye. The dilated funduscopic exam was unremarkable in both eyes. The patient was diagnosed with bilateral dry eye disease, blepharitis, and cataracts. Because cataract in the right eye was visually significant, cataract surgery was subsequently scheduled.

Prior to surgery, the patient was on daily antihistamines as MCAS treatment at 30 mg of cetirizine per day. To avoid potential inflammation peri-operatively in a patient with underlying MCAS, the decision was made by the managing team to pre- and post-operatively treat the patient with anti-inflammatory agents, including antihistamines and systemic corticosteroids. Pre-procedurally, 25 mg of intravenous (IV) diphenhydramine was administered 30 minutes prior to initiation of the procedure. Tropicamide 1% (10 mg/mL), a muscarinic antagonist used intraprocedurally to dilate the pupil (mydriasis) and paralyze pupil accommodation (cycloplegia), was instilled 3 times for dilation in the right eye; phenylephrine, an alpha-adrenergic agonist also typically used for mydriasis, was not used to prevent possible drug-related inflammation. At the conclusion of the surgery, the right eye was thoroughly irrigated, and a subconjunctival injection of dexamethasone (2 mg/ml) was administered. The right eye appeared slightly injected with a clear cornea, which is consistent with standard postoperative appearance. In the post-anesthesia care unit, he was prescribed methylprednisolone (tapered over six days), and prednisolone acetate 1% eye drops four times daily (standard of care) to be tapered over a period of four weeks to prevent and reduce inflammation.

**Fig. 1:**
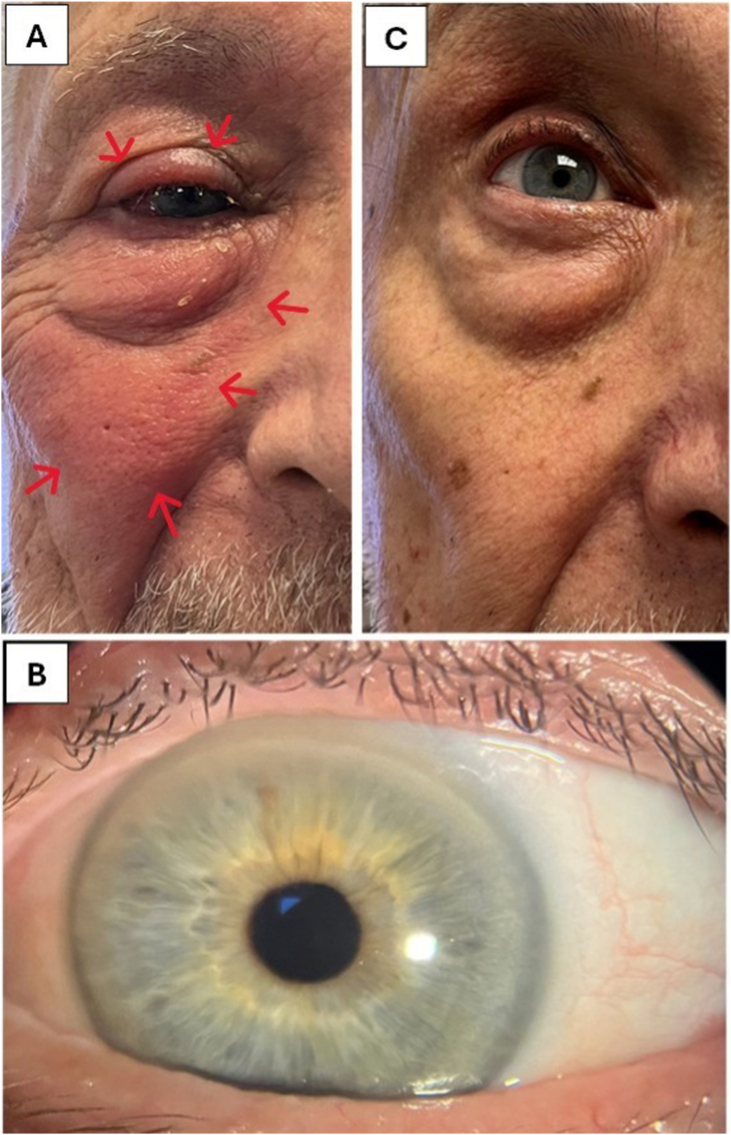
Unusual periocular inflammation associated with mydriatic eyedrops in a patient with Mast Cell Activation Syndrome (MCAS). (A) After dilatation with tropicamide, the patient exhibited periocular edema, erythema (arrows), and warmth on the first postoperative day despite preventive therapy. (B) At the 2-week follow-up with postoperative tapered methylprednisolone, and prednisolone acetate 1% eye drops, the patient had a clean cornea with 20/20 visual acuity and (C) resolved right eye periocular inflammation.

Despite anti-inflammatory preventive measures, on postoperative day 1 (POD1), approximately 15 hours after surgery, the patient presented with periocular (upper and lower) swelling, erythema, and warmth in the right eye (Fig. 1 A). No additional dilating drops, including tropicamide, were administered on POD1. However, the visual acuity of the right eye was 20/20, with a clear cornea, white and quiet conjunctiva, an adequate tear lake, and a moderate tear break-up time of 7 seconds. The cataract surgery was uneventful, suggesting that intraocular inflammation or postoperative infection was not the cause of the periorbital inflammation. The patient was counseled to continue the systemic and topical ophthalmic steroids. At the 2-week follow-up, the patient exhibited a clear cornea (Fig. 1 B) with visual acuity of 20/20 and resolved periocular inflammation in the right eye (Fig. 1 C). He remained symptom-free, and the periocular inflammation completely subsided at the 1-month follow-up (Timeline graphic summary in Table 1).

**Table 1:** Timeline graphic summary of clinical course.

Time Point	Exposures/Interventions	Clinical Findings
**Pre-op (baseline)**	Daily cetirizine 30 mg for MCAS	No periocular inflammation
**POD0 – Pre-procedure**	IV diphenhydramine 25 mg (30 min pre-op)	Asymptomatic
**POD0 – Intraoperative**	Tropicamide 1% × 3 instillations; phenylephrine not used; balanced salt solution irrigation	smooth intraoperative course without complication
**POD0 – End of surgery**	Thorough ocular irrigation; subconjunctival dexamethasone 2 mg/mL	Mild conjunctival injection, clear cornea (expected postoperatively)
**POD0 – PACU**	Oral methylprednisolone taper initiated; prednisolone acetate 1% eye drops QID	No periocular swelling
**POD1 (∼15 hours post-op)**	No additional dilating drops administered	Periocular upper and lower lid swelling, erythema, warmth; VA 20/20; clear cornea; white and quiet conjunctiva; no intraocular inflammation
**POD1 Management**	Continued systemic and topical corticosteroids	Stable ocular exam
**POD14 (2-week follow-up)**	Ongoing steroid taper	Resolution of periocular inflammation; clear cornea; VA 20/20
**POD30 (1-month follow-up)**	Completion of postoperative medications	Completely symptom-free

## 3 Discussion

Mast cells are known to be involved in allergic reactions, but they continue to be among the least understood components of the multifunctional cells engaged in innate and acquired immunity. This is the one of the few case reports documenting periocular inflammation associated with mydriatic eye drops in a patient with MCAS. Although genetic testing for hereditary alpha-tryptasemia was not performed and therefore this condition cannot be excluded, our case fulfills the definition of MCAS as diagnosed by characteristic episodic symptoms (chronic diarrhea, ocular swelling), an elevated serum tryptase measurement, and an adequate response to therapies targeting mast cell mediators.^5^ His skin lesions were negative for mast cells, which makes mastocytosis unlikely. This case report highlights the need for ophthalmologists to be aware of patients with MCAS when standard of care mydriatic agents are used, especially when undergoing cataract surgery, as these patients may experience severe and potentially life-threatening systemic reactions related to the release of mast cell mediators.^6^

The pathophysiology of mast cells is an area of research that is becoming more well defined. Mast cells can be triggered and activated by known or idiopathic triggers, and the release of their mediator granules, including histamine and tryptase, can lead to the symptoms observed in an allergic reaction, including, but not limited to, cutaneous flushing, swelling, hives, wheezing, with the most severe reactions leading to anaphylaxis.^7^ Mast cells are also the causative cells in MCAS, a disease in which mast cells cause episodic symptoms like those seen in allergic reactions. Mast cells can be triggered by exogenous factors, including drugs and excipients (Table 2), such as preservatives, stabilizers, solvents or by unknown triggers (idiopathic).^8^, ^9^, ^10^ Benzalkonium chloride (BAC), a common preservative in many ophthalmic drops including tropicamide, is a known trigger of mast cell–mediated reactions. Drug-induced mast cell activation can be IgE-mediated and non-IgE-mediated mechanisms (e.g., via the Mas-related G protein-coupled receptor X2, MRGPRX2 receptor on mast cells).^11^ Some medications, including opioids, neuromuscular blocking agents, vancomycin, fluoroquinolones, and nonsteroidal anti-inflammatory (NSAIDS), may trigger mast cell activation in susceptible individuals with MCAS,^12^ although many patients tolerate these agents well. M2 Muscarinic receptors are expressed on mast cells, and muscarinic receptor antagonists such as tropicamide may theoretically exert inhibitory mast cell activation, but their effects specifically in patients with MCAS remain poorly understood.^13^ This is one of the first case reports detailing a post-operative reaction to excipient or tropicamide itself, a muscarinic antagonist, in a patient with MCAS.

**Table 2:** Comprehensive exposure table.

Category	Agent	Concentration/Dose	Brand (if known)	Preservative
Periocular antiseptic	Povidone-iodine	5%	Betadine®	None
Drape adhesives/tapes	Surgical tapes	–	–	–
Ocular surface irrigation solution	Balanced salt solution	Standard	Alcon	None
Ophthalmic viscoelastic device (OVD)	Dual-Viscot, Viscot, Healon	Standard	Alcon/Johnson & Johnson	None
Topical Anesthetics drops	Proparacaine 0.5%	0.50%	Alcaine®	None
Sedative medication	Midazolam	1–2 mg IV	–	–
Topical antibiotics	Moxifloxacin 0.5%	0.50%	Vigamox®	None
Topical mydriatics drops	Tropicamide 1%, Phenylephrine 2.5%	1%/2.5%	Various	Benzalkonium chloride (BAC)
Topical Postoperative drops	Prednisolone acetate 1%, Ketorolac 0.5%	1%/0.5%	Pred Forte®/Acular®	Benzalkonium chloride (BAC)

Previous reports on ocular manifestations in patients with MCAS are limited. In a French single-center cohort of 21 patients with mastocytosis, a mast cell disease distinct from MCAS, dry eye disease and blepharitis were frequently reported.^3^ Our patient also presented with both dry eye disease and blepharitis, however, interpretation of these findings are challenging due to the high prevalence of dry eye disease and blepharitis in the general population. Nevertheless, they may represent inflammatory ocular surface manifestations of MCAS, such as eye irritation, and could have been part of the patient's longstanding multisystem disease.^2^ The comorbidity of eye disease presents uncertainty on whether the separate ocular conditions are related to MCAS or if they are simply coexisting diseases. Additional cases have reported ocular presentations in patients with mast cell disease (mastocytosis) including solitary eyelid mastocytomas, keratoconjunctivitis, choroidal mast cell infiltration, conjunctival chemosis, and exophthalmos.^3^ In a French case-control study, 60% of patients diagnosed with mastocytosis reported experiencing 'ocular discomfort,' and among these patients, 15% classified their discomfort as severe or intolerable.^14^ Reviewing drug-related allergic ocular reactions further, contact allergic reactions to atropine, a competitive, reversible antagonist of muscarinic receptors, and other mydriatic agents have been infrequently reported in MCAS patients or in the general population. In a Spanish study of 37 healthy patients who had adverse reactions to mydriatic eyedrops including symptoms of ocular pruritis, lacrimation, edematous erythema, blepharitis lasting several days, phenylephrine was the most common causative agent the most often (93.5%).^15^ In practice, tropicamide 1%, a muscarinic antagonist, when administered concurrently with phenylephrine 2.5%, an alpha-adrenergic agonist, demonstrates a generally well tolerated safety profile with allergic reactions rarely, if ever, observed, when administered ophthalmically.

The differential diagnosis for postoperative periocular erythema and swelling includes infectious cellulitis, allergic contact dermatitis, angioedema or vascular edema, postoperative inflammatory edema, and drug-related hypersensitivity reactions. Infectious cellulitis was considered unlikely given the absence of fever, pain, tenderness, visual deterioration, purulent discharge, or signs of intraocular inflammation, as well as the rapid improvement with continued anti-inflammatory therapy alone. Postoperative edema typically presents immediately after surgery and improves within days, whereas this patient developed localized periocular inflammation with delayed onset (15 hours) and complete resolution over weeks. Angioedema was considered less likely due to the absence of systemic involvement. Allergic contact dermatitis or mast cell–mediated hypersensitivity, potentially triggered by perioperative medication exposures, remains a plausible explanation in the context of the patient's underlying MCAS.

Taking into consideration our patient's history of MCAS, he was pretreated with IV antihistamines, received only intraprocedural tropicamide without atropine or phenylephrine, was administered intraoperative subconjunctival dexamethasone injection after thorough irrigation, and finally received postoperative solumedrol dose pack and dexamethasone eyedrops. Despite these extensive safety measures, our patient experienced periocular allergic inflammation. Although mast cells may trigger inflammation throughout the body, direct evidence linking dilating drops or associated preservative to periocular inflammatory skin reactions in MCAS patients remains to be explicitly established. The periocular inflammatory reaction shows a suggestive association with pharmacologic pupil dilation and may reflect either a response to the mydriatic agent or preservatives in the ophthalmic preparation that triggered mast cell–mediated inflammation. Indeed, the findings support a suggestive association rather than a definitive causal relationship. In general, however, exposure to multiple ophthalmic medications containing preservatives may cumulatively trigger postoperative inflammatory flares in patients with MCAS, and discontinue these agents usually allow the reaction to resolve. The patient's prior episodes of postoperative periocular erythema and swelling following dilating drops, although the specific agents could not be identified and biochemical confirmation such as serum tryptase levels was lacking, support an inferred role of mast cell mediator release based on the patient's prior multidisciplinary diagnosis, characteristic episodic symptoms, and clinical response to therapies targeting mast cell mediators. Overall, both mechanisms - direct drug effect and excipient-triggered mast cell activation - are plausible with the known behavior of MCAS.

Based on the known behavior of MCAS, we provide a practical checklist recommending that ophthalmologists follow a perioperative strategy, including histamine 1 or 2 (H1/H2) receptor blockade, leukotriene antagonists if indicated, use of preservative-free drops, the lowest effective mydriatic dose, and careful ocular surface irrigation. If repeat dilation is required, prior reactions should be reviewed, and alternative strategies considered. With ocular findings associated with MCAS are now being reported, it appears that patients likely suffer from more eye symptoms and greater related disability than previously thought; hence, ocular disease associated with MCAS may be underdiagnosed. The characteristics and related factors of ocular manifestations in MCAS, such as in our case, have not been adequately assessed or described. This oversight underscores the need for further awareness among healthcare providers and research to understand the broad spectrum of ocular presentations associated with MCAS. Increased awareness among ophthalmologists may facilitate earlier recognition of MCAS and appropriate referral for systemic evaluation and management.

## CRediT authorship contribution statement

**Anny M.S. Cheng:** Writing – review & editing, Writing – original draft, Validation, Supervision, Methodology, Formal analysis, Conceptualization. **Aishah Ali:** Writing – review & editing, Writing – original draft, Validation, Supervision, Methodology, Formal analysis. **John T. LiVecchi:** Writing – original draft, Supervision, Software, Project administration. **Hailey Mair:** Writing – original draft, Software, Project administration. **Elizabeth S. Yang:** Writing – original draft, Software, Project administration. **Jade Gieseke Guevara:** Writing – review & editing, Writing – original draft, Supervision, Methodology, Formal analysis, Conceptualization.

## Patient consent

Consent to publish this case report has been obtained from the patient in writing.

## Data statement

All data analyzed during this study are included in this article. All data can be provided upon request to corresponding author.

## Financial disclosure

No authors have any proprietary interest in this study.

## Declaration of competing interest

The authors declare that they have no known competing financial interests or personal relationships that could have appeared to influence the work reported in this paper.
